# Path Analysis of the Risk of Low Birth Weight for Multipara

**DOI:** 10.5812/ircmj.3592

**Published:** 2013-06-05

**Authors:** Amir Hamta, Ali Reza Khalilian, Roya Farhadi, Hossein Ranjbaran

**Affiliations:** 1 Department of Biostatistics and Community Medicine, Mazandaran University of Medical Sciences, Mazandarana, IR Iran; 2 Department of pediatrics, Mazandaran University of Medical Sciences, Mazandarana, IR Iran; 3 Mazandaran University of Medical Sciences, Mazandarana, IR Iran

**Keywords:** Path Analysis, LBW, Multipara

## Abstract

**Background:**

LBW rate is one of the most important health indices in every society. It reveals mothers and their new-born infants' health.

**Objectives:**

Our aim, in the present paper, was to present a new statistical framework for analysis based on path analysis techniques.

**Patients and Methods:**

A prospective study was conducted in two maternity wards, (privet & governmental hospital) in Sari, Iran. In this research a check-list containing 25 questions about mother’s demographic information and her new-born infant was prepared. Every new born infant who was born weighing less than 2500 g was entered in our study and just next the newborn infant who was normal all of his/her information use to be taken too, (n = 190). Path analysis, an extension of the regression model, was used in this study.

**Results:**

Obviously exactly half of the infants were LBW, and the remainder were normal. There were 97 boys and 93 girls. The percentage of IUGR among mothers who had preterm delivery was 19, while this percentage for mothers who had term delivery was 11.5 (P value = 0.167). LBW infants were 36.7% unexpected, while this percent for normal infants was 15.5 (P value < 0.001). Preterm delivery has a significant and direct effect on LBW (p value < 0.001), and its positive sign of path coefficient shows that if it occurs, the probability of LBW will increase, the second important was IUGR, the results showed unexpected pregnancy had direct effect on LBW but this wasn’t significant (P value = 0.292).

**Conclusions:**

By preventing unnecessary termination of pregnancy and keeping fit, the chances of LBW can be reduced.

## 1. Background

Low birth weight (LBW) is significantly related to mortality (1). Consequences of LBW especially in third world countries is superabundant (2). LBW rate is one of the most important health indices in every society (3). It indicates the mother’s and her new born infant’s health (4). Approximately the incidence of LBW is 16% globally (LBW < 2500 gr) (5) and more than 95 percent of LBW infants are born in third world countries. Seventy- two percent of LBW infants are born in Asia; although there are large differences in WHO statistics for Asian regions and its sub-regions, it is estimated that there are 8% of LBW infants in Eastern Mediterranean region (6). Many studies have accounted for the risk factors for preterm delivery and for LBW (7-10) as well as for neonatal outcomes. The short-term outcomes on ante partum, labor and postpartum care of LBW infants have not yet been properly investigated. There is no general consensus that LBW fetuses are more susceptible to fetal distress than normal weight and that there are differences in labor, labor induction and mode of delivery between them and that newborns are prone to lower Apgar scores. Our aim in the present paper was to present a new statistical framework for analysis based on path analysis techniques, in order to distinguish between direct and indirect factors affecting the risk of LBW. Path analysis was originally introduced by wright (11), has been popularized by Li in population genetics (12) and used by Duncan in the social science (13).

## 2. Objectives

Our aim in the present paper was to present a new statistical framework for analysis based on path analysis techniques.

## 3. Patients and Methods

A prospective study was conducted during 2010 to 2011 in 2 maternity wards of the main general and teaching hospitals of Sari, Iran. Participants were selected from both private and governmental hospitals. The ethics committee of Mazandaran University of Medical Sciences approved the study. In this research a check-list containing 25 questions about the mother’s demographic information and her new born infants was prepared. Every new born infant who was born less than 2500 g was entered in our study and just next new born infant who was normal all of his/her information was taken too. Mothers who had severe diseases such as cancer, and diabetes and only one pregnancy were included in this study. Scales, which measured weight in both hospitals were the same and were adjusted every day by the supervisor of the ward.

Path analysis, an extension of the regression model, was used in this study. A path coefficient, represented as pij, is a standardized regression coefficient indicating the direct effect of variable i on variable j. Accordingly, in a multivariate regression system, it is a partial regression coefficient controlling for other prior variables. Basically, whenever a causal, rather than spurious or coincidental, correlation among a set of variables is suspected, the path analysis is strongly suggested to be done, especially when there is a possibility to sort out the sequence of variables or when it is necessary to distinguish the spurious effect by an intervening factor from the observed relationships (14) The path analysis has several advantages. First of all, it allows for effect decomposition because the total causal affects the sum of the values of all paths from i to j. The indirect effect, measuring the effect of the intervening variables, is the total causal effect minus the direct effect. A second advantage of path analysis is shown by path diagram, which explains the hypothetical causality graphically. It runs through from the left to the right, with the exogenous variables or independent variables on the extreme left and the dependent or endogenous variables (the model tries to explain) on the right side. A straight line represents the relationship between the two variables. As Kline (15) recommended, 10 to 20 times as many cases as parameters were needed to assess significance. If the ratio of the number of cases to the number of parameters was below 5, there would be great possibility for the model estimation to be unstable (16). In this study we had 9 observed variables so based on Kline’s suggestion, 189 samples were eligible and all of them, which were multipara were taken. For analyzing data LISREL 8.8 (Scientific Software International, Inc; USA) was used.

## 4. Results

In the present study, information from 190 mothers and their new-born infants was collected. Obviously, half of the infants were LBW, and the remainders were normal. There were 97 boys and 93 girls. 30.7 percent of mothers had preterm delivery and 13.8 of mothers had Intrauterine Growth Retardation (IUGR). The percentage of IUGR among mothers who had preterm delivery was 19, while this percentage for mothers who had term delivery was 11.5 (P value = 0.167). 75.7% of cases had intentional pregnancies and the rest of had unexpected pregnancies. LBW infants were 36.7% unexpected, while this percent for normal infants was 15.5 (P value < 0.001). Mothers 61.9% had 2 pregnancies , 22.8% had three pregnancies, 10.1% had four pregnancies and the rest of them had more than 4 pregnancies; Table 1 shows mean and standard deviation of various variables separately for LBW and normal infants. 

**Table 1.** The Demographic Characteristics of Mothers of Infants Under Study Based on the Weight at Birth

	Mothers (Total), Mean ± Standard Deviation	Mothers of LBW Infants, Mean ± Standard Deviation	Mothers of Natural Born Babies, Mean ± Standard Deviation	P value
**Age of Marriage, y**	19.66 ± 3.84	18.03 ± 3.382	20.84 ± 3.74	< 0.001
**Age, y**	29.413 ± 4.42	28.241 ± 4.03	30.25 ± 4.51	< 0.01
**Gestational age, Week**	36.61 ± 3.67	33.79 ± 3.92	38.64 ± 1.55	< 0.0001
**BMI**	29.72 ± 4.86	27.47 ± 4.73	31.34 ± 4.29	< 0.01
**First pregnancy age**	22.23 ± 3.714	21.19 ± 3.64	22.97 ± 3.6	< 0.001
**Distance between last pregnancy & recent pregnancy, y**	5.64 ± 2.55	4.79 ± 2.38	6.24 ± 2.5	< 0.01
**Birth weight of last child, gr**	3000.49 ± 705.29	2796.63 ± 751.1	3146.89 ± 634.38	< 0.01
**Infant weight**	2740.56 ± 818.423	1925.82 ± 513.31	3325.68 ± 374.32	< 0.0001
**Mothers' BMI**	29.72 ± 4.86	27.48 ± 4.73	31.34 ± 4.29	< 0.01

Since the correlation matrix, beside theoretical concepts, is a great full for achieving Path analysis model, this was calculated (Table 2).

**Table 2.** Correlation Coefficients Among the Variables

	Age	Marriage age	First Pregnancy age	Distance Between Last Pregnancy and Recent Pregnancy, y	Weight of Last Child	Gestational age	BMI	Infants’ Weight
**Age**	1							0.223^b^
**Marriage age**	0.57 8^b^	1						0.365^b^
**First Pregnancy age**	0.568^b^	0.906^b^	1					0.265^b^
**Distance Between Last Pregnancy and Recent Pregnancy, y**	0.541^b^	0.049	-0.018	1				0.224
**Weight of Last Child**	0.093	-0.005	-0.068	0.155^a^	1			0.235^b^
**Gestational age**	0.099	0.297^b^	0.218^b^	0.130	0.093	1		0.793^b^
**BMI**	0.206^b^	0.070	-0.004	0.220^b^	0.205^b^	0.146^a^	1	0.256^b^
**Infants’ Weight**	0.223^b^	0.365^b^	0.265^b^	0.224^b^	0.235^b^	0.793^b^	0.256^b^	1

^b^Correlation is significant at 1% level

^a^Correlation is significant at 5% level

**Figure 1.**
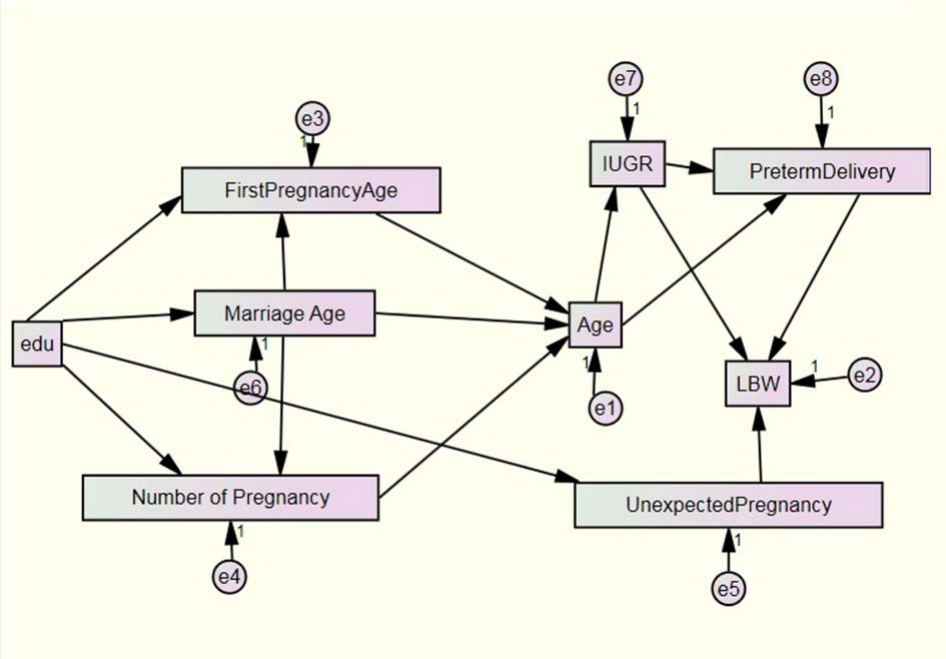
Input Path Diagram

As it seen in Table 2 there are significant correlations between the majority of variables. Before anything else, binary variables by using logic link function were transferred into continuous variables and their predicted probabilities were calculated. Since we have to pay attention to the principle of parsimony for the statistics of suitable sub collections and the selection of a pattern in drawing the path diagram (Because the lower the number of predicting variables, the simpler the control of dependable variables) ( 17 ), there is no need for the model to have an extensive variable and it is possible to have more than enough variables in the model that is not possible to be tested ( 18 ) in the first stage path diagram1 was considered as a input path diagram Figure 1. 

**Figure 2.**
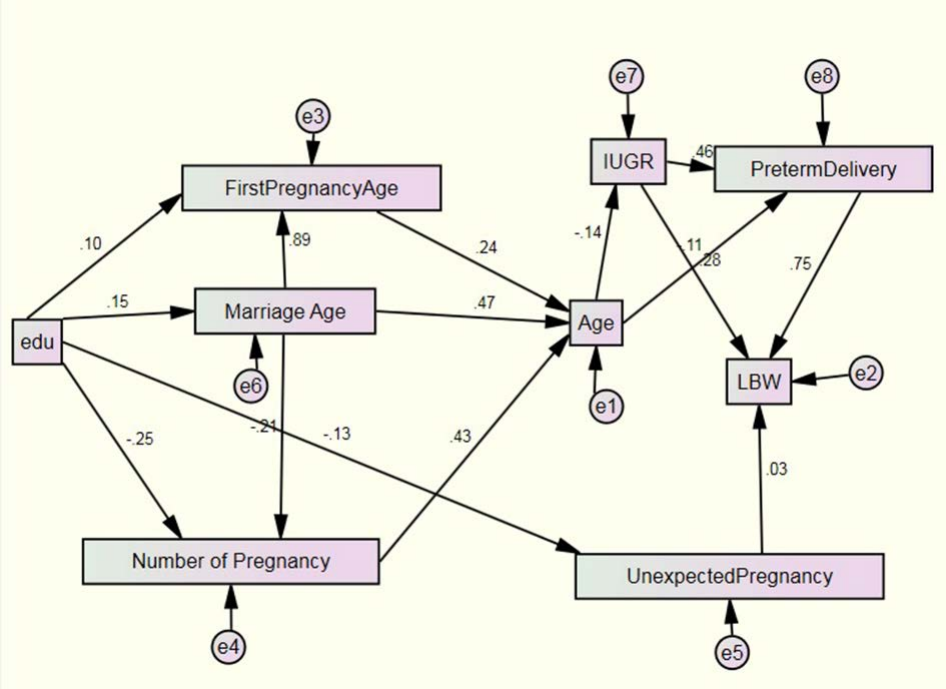
Output Path Diagram

In the current input path diagram there are 17 variables, e _1 _s are unobserved exogenous variables and only education is an observed endogenous variable. In the next stage, output path diagram was reached in Figure 2 and path coefficients, written above of each path, were calculated. 

The straight lines show the direction of the path from the exogenous variables towards the indigenous variables. In the last decade, a lot of statistics were presented for testing the model ( 18 ); there are 30 indices for model fit judgment ( 19 ) and the majority of them are reported in LISREL. Unfortunately there are no preferences for using them and it depends on sample size, normality of data, estimation methods, and complexity of model and so on. They are categorized into three categories: 1) absolute fit indices, 2) comparative fit indices and 3) parsimonious fit indices. Absolute fit indices answer this question whether error variance after fitting the model is considerable or not and they do not have any base line. Comparative fit indices are used for comparing models and parsimonious fit indices show whether model is economic or not. Table 3 shows model fit indices for the considered model. 

**Table 3.** Fit Indices for Path Model

Index	RMR	RFI	TLI	IFI	CFI	NFI	PNFI	PCFI	Hoelter
**Value**	0.055	0.965	1	1	1	0.979	0.571	0.583	183

For the RMR which is the absolute fit index, the closer value to zero shows a better fit ( 20 ). NFI, TLI, RFI, IFI and CFI, which are comparative fit indices show that this model is fitted very well. PNFI and PCFI reveal that the model is economical and Hoelter shows our sample size was large enough. Table 4 shows direct, indirect and total effect calculated by path analysis. In the next stage the direct, indirect and total effect of variables on each other were calculated (Table 4). 

**Table 4.** Direct, Indirect and Total Effect Obtained by Path Analysis Model

Relationship	Direct Effect	Indirect Effect	Total Effect
**Marriage age on LBW**	0	-0.098	-0.098
**First pregnancy age on LBW**	0	-0.040	-0.040
**education on LBW**	0	-0.005	-0.005
**Number of pregnancies on LBW**	0	-0.071	-0.071
**IUGR on LBW**	0.284	0.342	0.626
**Age on LBW**	0	-0.166	-0.166
**Unexpected pregnancy on LBW**	0.031	0	0.031
**Preterm delivery on LBW**	0.746	0	0.746

Preterm delivery had a significant direct effect on LBW (p value < 0.001), and its positive sign of path coefficient shows that if it occurs, the probability of being LBW will increase. The second important entity was IUGR, the results showed unexpected pregnancy had a direct effect on LBW but this wasn't significant (P value = 0.292).

## 5. Discussion

The purpose of this study was to identify and confirm the direct and indirect effect of factors on LBW. A rise of LBW rate in a country increases neonatal mortality and also infant mortality rate, which is an important indicator of the level of public health in the country (21). LBW infants have a lower IQ than normal ones (22). The effect of smoking during pregnancy on LBW was examined. No effect of multipara was seen. We hypothesize that in this sample the absence of an effect was seen because of the low rate maternal smoking during pregnancy. In addition, mothers reported less consistently on smoke than on other variables.

Path analysis is superior to ordinary regression analysis as it provides and explains both casual relations and the relative importance of alterative paths on influence (23). The path coefficient shows that preterm delivery has the largest direct effect and explains the greatest variance on LBW. An increase of one standard deviation of pre-term delivery produces and increases LBW by 0.75 standard deviations. Khalilian et al. in a survey showed that preterm delivery has a significant effect on LBW (24). Also IUGR and unexpected pregnancy had a direct effect on LBW by 0.28 and 0.03, respectively. The final path model shows that age had a significant negative indirect effect and IUGR had a significant positive indirect effect on LBW. Also marriage age, first pregnancy age, education, number of pregnancies and unexpected pregnancy had a non-significant indirect effect on LBW. By preventing unnecessary ending of pregnancy and keeping fit the chances of LBW can be reduced.

Univariate analysis revealed factors related to low birth weight, including, marriage age, mother’s age, gestational age, BMI, first pregnancy age, distance between last pregnancy and recent pregnancy, birth weight of last child, and finally infant weight and obviously all of them had a positive effect on weight of new born infants, while unexpected pregnancy had a negative effect and caused LBW. Somehow these findings are in agreement with those reported by some studies about risk factors of LBW (2, 25). In the multivariate analysis only a few variables had a significant effect, because their influence was considered in the other variables included in the model, thus paying attention to multivariate analysis besides univariate analysis can prevent false interpretations. By path analysis which is one of the advanced multivariate methods not only the effect of each variable on dependent variables can be measured, but also the effect can be separated in to direct and indirect categories.

### 5.1. Suggestions

Unfortunately path analysis cannot demonstrate causality, and cannot be used in explanatory situations thus finding some method that has path analysis’ properties and can be used for explanatory purposes, would be very useful. Because of path analysis prerequisite, which are variables used in the model, must have at least interval scale finding some method can be used when variables have nominal scale is beneficial. Using Bayesian path analysis for the next investigation is strongly recommended.
